# Discovery of a Short‐Chain Dehydrogenase from *Catharanthus roseus* that Produces a New Monoterpene Indole Alkaloid

**DOI:** 10.1002/cbic.201700621

**Published:** 2018-03-22

**Authors:** Anna K. Stavrinides, Evangelos C. Tatsis, Thu‐Thuy Dang, Lorenzo Caputi, Clare E. M. Stevenson, David M. Lawson, Bernd Schneider, Sarah E. O'Connor

**Affiliations:** ^1^ Department of Biological Chemistry John Innes Centre Norwich Research Park Norwich NR4 7UH UK; ^2^ UMR DIADE Institut de Recherche pour le Développement BP 64501 34394 Montpellier France; ^3^ Max Planck Institute for Chemical Ecology Hans-Knöll-Strasse 8 07745 Jena Germany

**Keywords:** alkaloids, biosynthesis, natural products, reduction, short-chain dehydrogenases

## Abstract

Plant monoterpene indole alkaloids, a large class of natural products, derive from the biosynthetic intermediate strictosidine aglycone. Strictosidine aglycone, which can exist as a variety of isomers, can be reduced to form numerous different structures. We have discovered a short‐chain alcohol dehydrogenase (SDR) from plant producers of monoterpene indole alkaloids (*Catharanthus roseus* and *Rauvolfia serpentina*) that reduce strictosidine aglycone and produce an alkaloid that does not correspond to any previously reported compound. Here we report the structural characterization of this product, which we have named vitrosamine, as well as the crystal structure of the SDR. This discovery highlights the structural versatility of the strictosidine aglycone biosynthetic intermediate and expands the range of enzymatic reactions that SDRs can catalyse. This discovery further highlights how a sequence‐based gene mining discovery approach in plants can reveal cryptic chemistry that would not be uncovered by classical natural product chemistry approaches.

## Introduction

**Scheme 1:**
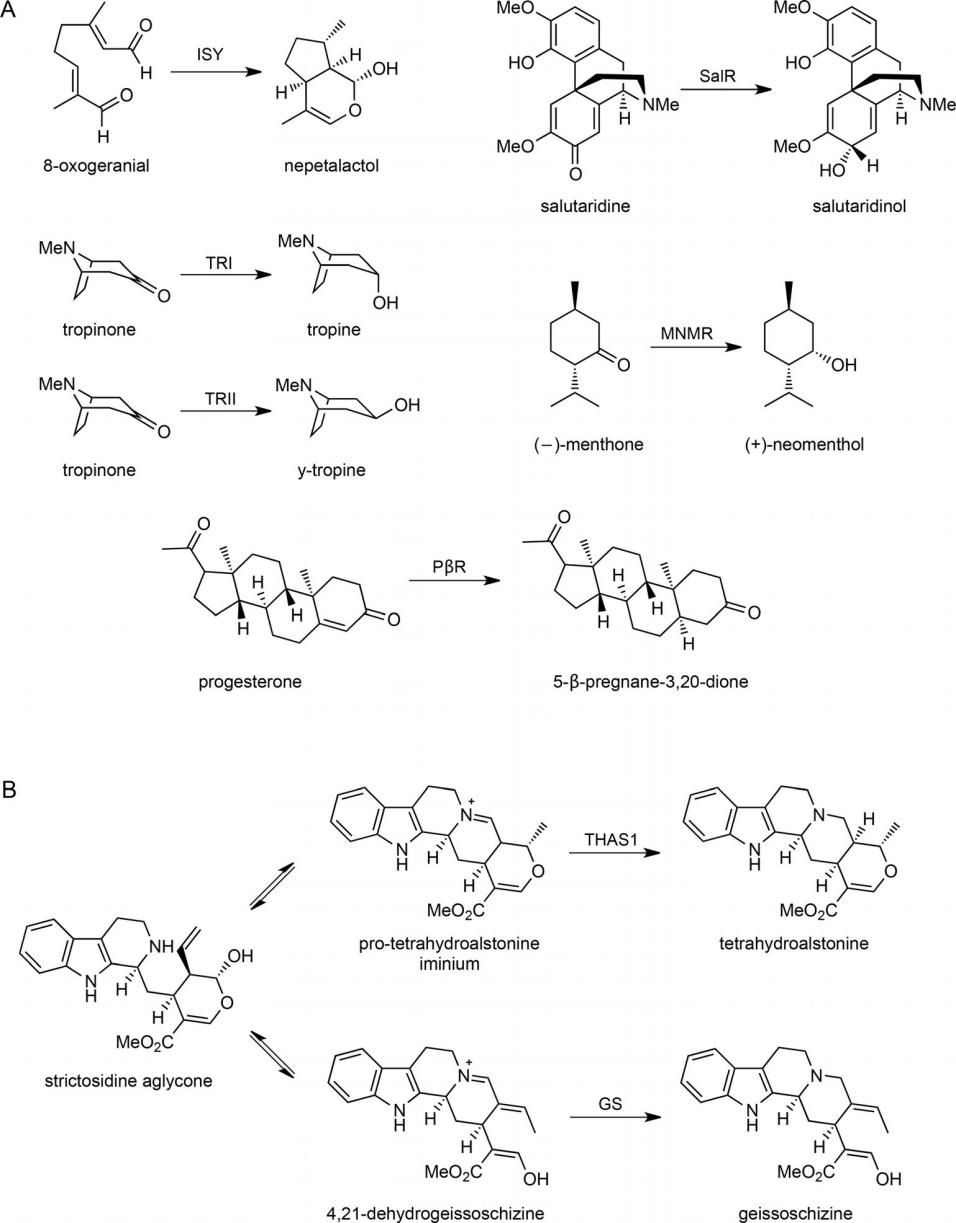
Reactions of SDRs in specialized metabolism. A) Previously identified SDRs in specialized metabolism include iridoid synthase (ISY), tropinone reductases I and II (TRI and TRII), salutaridine reductase (SalR), menthone reductase (MNMR) and progesterone 5‐β‐reductase (PβR). B) Strictosidine aglycone is a central biosynthetic intermediate of MIA biosynthesis that can undergo reduction to form various alkaloid backbones. THAS1 and GS are previously discovered medium‐chain alcohol dehydrogenases.

The short‐chain dehydrogenase/reductase (SDR) family is of ancient origin, and is found throughout the kingdoms of life, including viruses.^1^, ^2^, ^3^ SDRs each have a Rossmann fold for NAD(P)^+^ binding, and the SDR active site is characterized by a conserved catalytic tyrosine residue, which is accompanied by a lysine residue to give the conserved motif YxxxK; frequently a serine or threonine unit serves as an additional active‐site residue to make a catalytic triad.^4^ SDR gene families are particularly large in plants^5^ and many SDRs have been recruited into specialized metabolic pathways (Scheme 1 A). The monoterpene indole alkaloid (MIA) pathway of *Catharanthus roseus* is composed of approximately 100 compounds, including the anticancer agents vinblastine and vincristine.^6^ During the biosynthesis of these alkaloids, 8‐oxogeranial undergoes a reductive cyclization to yield nepetalactol through the action of the enzyme iridoid synthase, a member of the SDR family^7^, ^8^, ^9^ (Scheme 1 A).

In addition to iridoid synthase, other SDRs could potentially participate in MIA biosynthesis in *C. roseus*. An important branch point of MIA biosynthesis is at the intermediate strictosidine aglycone, which can be reduced to form a variety of different structures^10^ (Scheme 1 B). To investigate whether *C. roseus* harbours an SDR that uses strictosidine aglycone as a substrate, SDR gene candidates that were co‐regulated with known MIA biosynthetic genes were selected for screening in in vitro biochemical assays. One candidate—Cro013448—was tested against strictosidine aglycone and produced a compound that did not correspond to any MIA authentic standard or to any compound detected in *C. roseus* leaf extracts. Here we report the structural characterization of this product, which has not been previously reported, as well as the crystal structure of the SDR. This discovery highlights an additional role of SDRs in alkaloid biosynthesis, thus demonstrating the range of enzymatic reactions that SDRs can catalyse, and highlights the structural versatility of the strictosidine aglycone substrate of the MIA pathway.

## Results and Discussion

### Cloning, heterologous expression and assay of Cro013448

As part of a screen to identify new reductases involved in MIA biosynthesis, the *C. roseus* transcriptome was searched for SDRs with high expression in tissues treated with methyl jasmonate, an elicitor of MIA production^11^ (Figure S1 in the Supporting Information). We were particularly interested in identifying SDRs that act at the strictosidine aglycone branching point of the pathway. The gene coding for Cro013448 (GenBank: KP411011.1) was highly expressed in methyl‐jasmonate‐induced seedlings (>100 fpkm). Cro013448 was expressed as a His_6_‐tagged fusion protein in *Escherichia coli* and was purified by nickel and gel filtration chromatography (Figure S2). To screen for enzyme activity strictosidine was first deglycosylated in situ with the enzyme strictosidine glucosidase (SGD).^12^ Then, the cofactor nicotinamide adenine dinucleotide phosphate (NADPH) and an aliquot of Cro013448 were added to the reaction mixture. Gratifyingly, the substrate was consumed and a new product appeared.

### Cro013448 product characterization

The LC‐MS chromatogram of the enzymatic product did not correspond to authentic standards of any available MIA. The product had an *m*/*z* of 371.1967, and also appeared to undergo dehydration (*m*/*z* 353) during mass spectrometry (Figure S3). The fragmentation pattern indicated that the compound contains an indole moiety, with the typical 144 fragment observed for other indole alkaloids.^13^ In addition, a fragment of 342, which could potentially arise from the loss of a CH_2_O functional group, was observed. We did not detect any other characteristic MS fragments corresponding to other structural MIAs’ scaffolds. This was unexpected in view of the intense research focused on MIA (bio)synthesis.

To verify that the reduction is not a spurious reaction, a homologue from a closely related medicinal plant (*Rauvolfia serpentina*)—Rse2785—was also cloned and tested. This enzyme also yielded the same unknown product as evidenced by LC‐MS analysis (Figure S4). This suggests a conserved function among related plant species.

**Figure 1:**
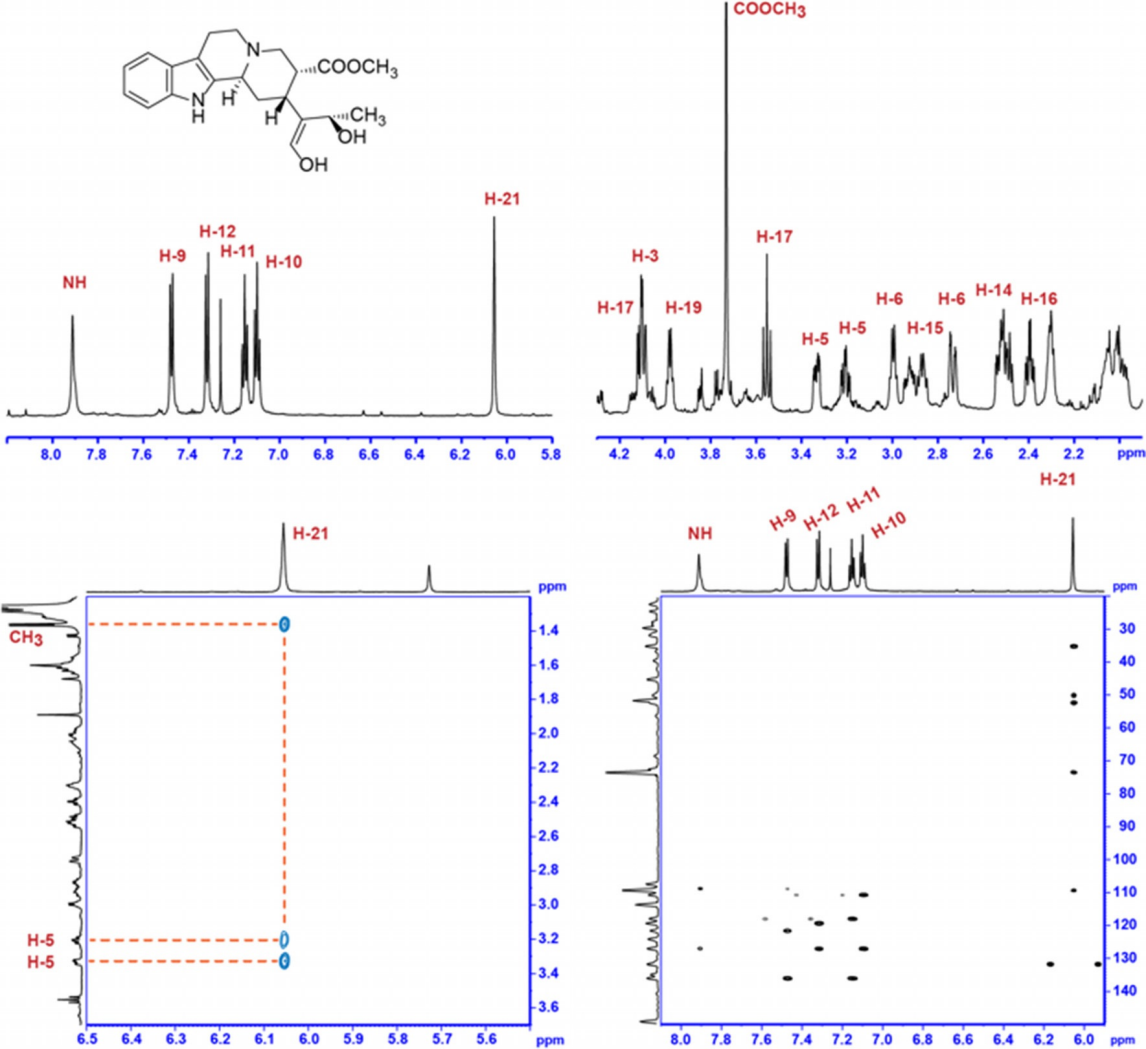
NMR spectra (700 MHz, CDCl_3_) of VAS (Cro013448) product. Top left and top right: sections of the ^1^H NMR spectrum. Bottom left: ROESY. Bottom right: HMBC. Strictosidine and vallesiachotamine numbering. The signal of 18‐CH_3_ (*δ*_H_=1.36 ppm) is not included in this figure. Ring D is indicated on the proposed chemical structure.

**Scheme 2:**
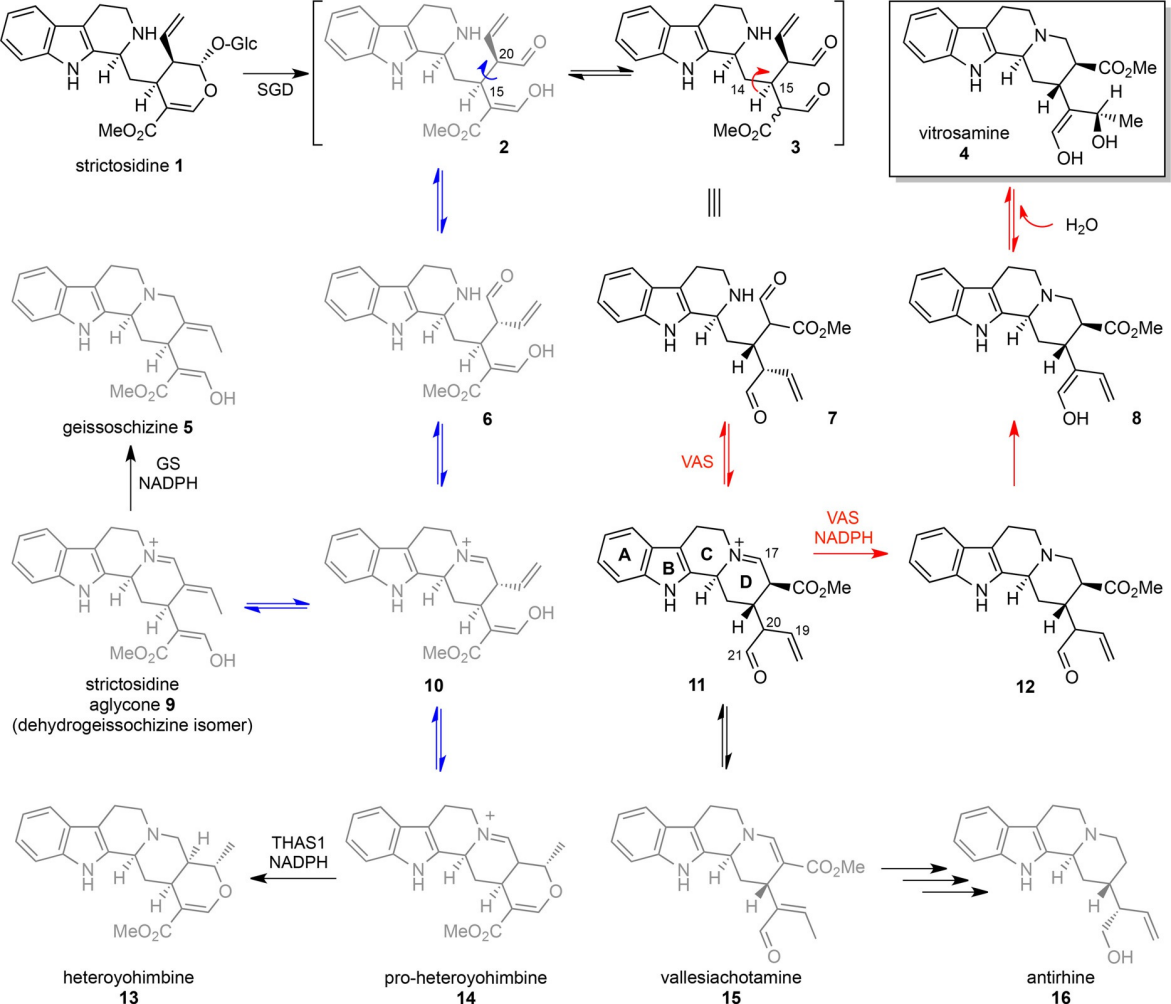
Rearrangement of strictosidine aglycone to give rise to different MIA structural classes. Rotation around the C‐15−C‐20 bond (blue arrow) and subsequent cyclization of ring D give rise to the heteroyohimbine and geissoschizine backbones. Rotation around the C‐14−C‐15 bond (red arrow) and subsequent cyclization of ring D yields the substrate of Cro013448, leading to vallesiachotamine (**15**), antirhine (**16**, previously observed natural products) and vitrosamine (**4**).

The Cro013448 product was produced on a milligram scale (Figure S5) and then analysed exhaustively by different NMR methods including ^1^H NMR, COSY, H2BC, HMBC and ROESY (Figures S6–S9, Table S1). The major product was isolated as the hydrated form. Correlations between carbon and hydrogen atoms are illustrated in Figures S8 and S9. The ROESY spectrum indicates that H‐21 is in proximity to the C‐5 hydrogen atoms. Correlations between the indole moiety and the remainder of the molecule through C‐2 and C‐7 were apparent. We observed a crosspeak between H‐21 and C‐2 that is also correlated with the NH moiety, thus confirming that these two regions of the molecule are connected through C‐2 and C‐7 (Figure 1). C‐21 existed in enol form, as observed in geissoschizine,^14^ and not the more stable aldehyde (Scheme 2, compounds **4** and **5**). Overall, the correlations for this product (Figure 1) suggest a structure that is similar to that of the natural MIA products vallesiachotamine (compound **15**, Scheme 2).

**Figure 2:**
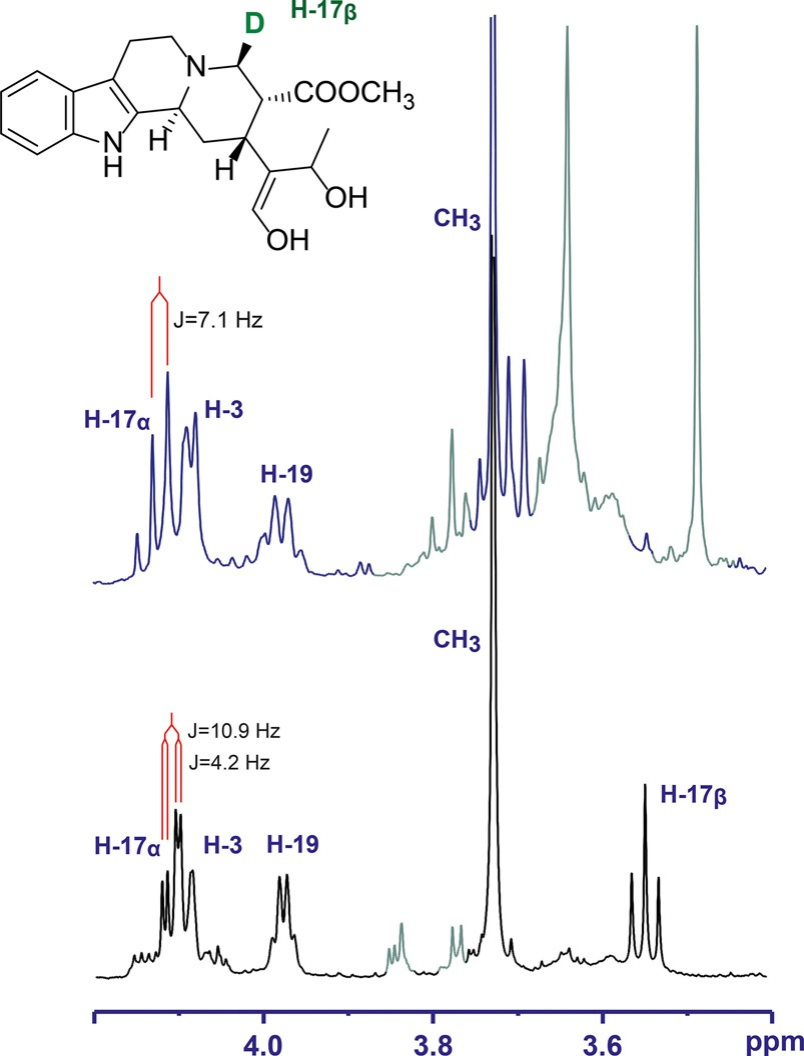
NMR spectra of deuterated product of VAS (Cro013448) (top) in comparison with non‐deuterated product (bottom). Signals from contaminants are coloured in grey.

To pinpoint the site of reduction, deuterium labelling was carried out with pro‐*S*‐deuterium‐labelled NADPD, because SDRs often transfer the pro‐*S* hydride of the cofactor. The MIA product generated with this labelled cofactor had an *m*/*z* of 372, which corresponds to one hydrogen atom being replaced by a deuterium atom. ^1^H NMR analysis indicated that H‐17β (*δ*=3.55 ppm) had disappeared, and the *J* coupling constant of H‐17α (10.9 Hz) shifted to 7.1 Hz (Figure 2). COSY analysis of the product showed only a crosspeak at *δ*=4.11 ppm, with the crosspeak at 3.55 ppm missing. Taken together, these data indicate that the cofactor hydride is added to C‐17 at the β position.

The Cro013448 product was given the name 19,21‐dihydro‐17‐dehydrovallesiachotamine, and the trivial name vitrosamine. This product does not correspond to any product found in *C. roseus* leaf extracts, and has not been reported to have been isolated from tissues from any plants. Cro013448 was renamed vitrosamine synthase or “VAS”.

### Crystal structure of VAS (Cro013448)

**Figure 3:**
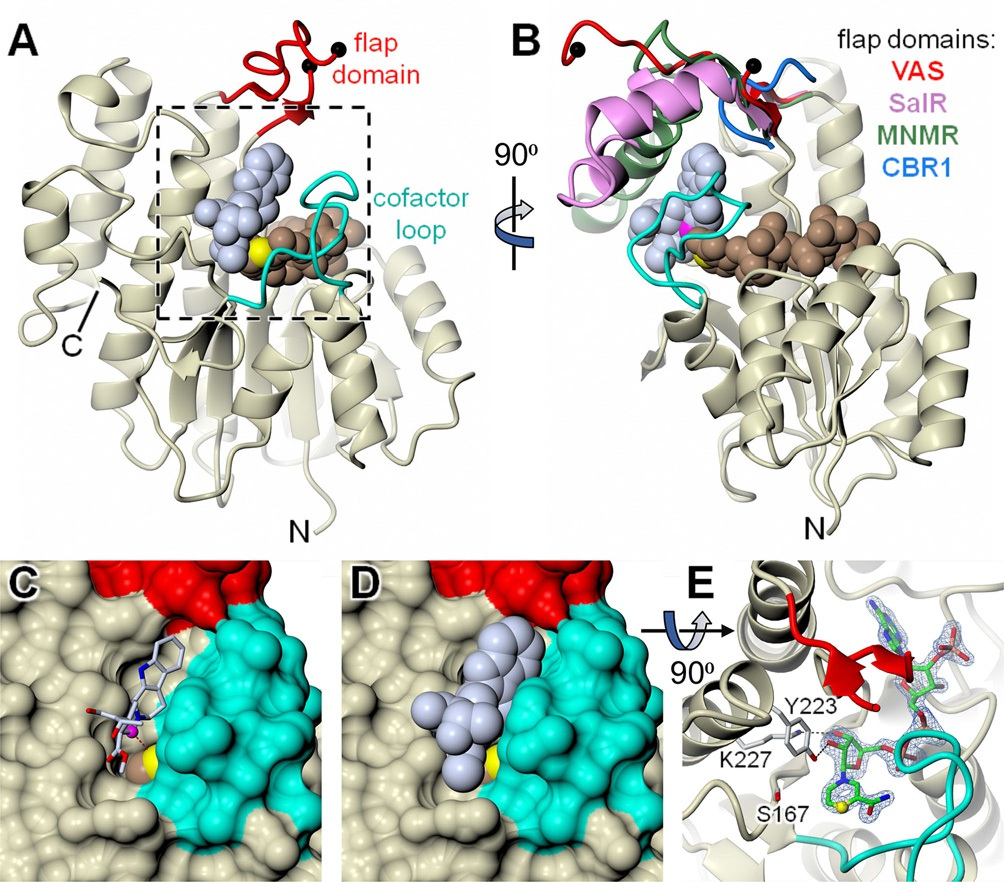
X‐ray structure of *C. roseus* vitrosamine synthase (VAS). A), B) Orthogonal cartoon representations of VAS, in which the core structure is coloured in magnolia and the flap domain and cofactor loop are shown in red and cyan, respectively. The black spheres indicate where there is a break in the backbone trace of the flap domain corresponding to a region that could not be resolved in the electron density. Also shown as van der Waals spheres are the bound NADP^+^ cofactor (light brown with C‐4 in yellow) and the docked substrate (ice blue). Additionally, in B), the flap domains of close structural homologues are shown after superposition of their full structures onto that of VAS. Specifically, these are *P. somniferum* SalR (pink; PDB ID: 3O26), *M. piperita* MNMR (green; PDB ID: 5L53) and human CBR1 (blue; PDB ID: 1WMA). However, in the last case the flap domain is merely a short loop connecting the equivalent of β4 and α4 in VAS. The full structural superposition is shown as a stereoview in Figure S10. C), D) The region highlighted in A) with the protein depicted as a molecular surface, with and without the docked substrate, respectively. Note that the cofactor loop almost entirely covers the nicotinamide “half” of the NADP^+^, except for the outer edge of the nicotinamide ring bearing C‐4, and that this is occluded by the docked substrate, which closely matches the dimensions of the active‐site pocket. E) The same region as D), but from above with the protein in cartoon. The NADP^+^ is coloured green (carbon atoms) but with the nicotinamide C‐4 atom in yellow. Also shown is the canonical SDR catalytic triad of Ser167, Tyr223 and Lys227, together with 1.55 Å resolution omit difference electron density for the cofactor (contoured at ≈5.0 *σ*). In this view, part of the flap domain was omitted for clarity.

To understand the mechanism of VAS better, the crystal structure was solved (Table 1 and Figure 3; PDB ID: 5O98). It closely resembles the structure of salutaridine reductase from *Papaver somniferum* (SalR; PDB ID: 3O26; i.e., the molecular replacement template^15^) and the recently determined structures of two SDRs from *Mentha piperita*:^16^ menthone‐neomenthol reductase (MNMR; e.g., PDB ID: 5L53) and isopiperitenone reductase (IPR; e.g., PDB ID: 5LCX; Figure S10). Crystallization of VAS was carried out with and without the oxidized cofactor NADP^+^. After solution of the structures it was clear that the apo enzyme active site also had a high occupancy of NADP^+^, thus indicating that VAS is purified from *E. coli* as a holoenzyme. Adjacent to the nicotinamide moiety of the cofactor is the canonical SDR catalytic triad consisting of Ser167, Tyr223 and Lys227.^4^, ^17^

**Table 1:** X‐ray data collection and refinement statistics for *C. roseus* vitrosamine synthase.

**Data collection**	
beamline	I02, Diamond Light Source, UK
wavelength [Å]	0.9795
detector	Pilatus 6M
resolution range^[a]^ [Å]	55.30–1.55 (1.59–1.55)
space group	*P*2_1_2_1_2_1_
cell parameters [Å]	*a=*58.83, *b=*61.02, *c=*162.03
total no. of measured intensities^[a]^	761 897 (28 422)
unique reflections^[a]^	85 013 (6006)
multiplicity^[a]^	9.0 (4.7)
mean *I*/σ(*I*)^[a]^	17.0 (1.5)
completeness^[a]^ [%]	99.5 (96.5)
*R*_merge_^[a,b]^	0.076 (1.120)
*R*_meas_^[a,c]^	0.090 (1.255)
*CC* ${}_{1/2}$ ^[a,d]^	0.997 (0.614)
Wilson *B* value [Å^2^]	28.8
**Refinement**	
resolution range^[a]^ [Å]	55.30–1.55 (1.59–1.55)
reflections: working/free^[e]^	80 707/4304
*R*_work_/*R*_free_^[a,f]^	0.169/0.190 (0.382/0.379)
Ramachandran: favoured/allowed/disallowed^[g]^ [%]	98.3/1.7/0.0
RMSD of bond distance [Å]	0.010
RMSD of bond angle [°]	1.50
no. of protein residues (ranges): chains A/B	282 (7–103; 115–299)/283 (7–107; 118–299)
no. of water molecules/NADP^+^ molecules	397/2
mean *B* factors: protein/water/NADP^+^/overall [Å^2^]	38.2/44.6/27.4/38.6
PDB ID	5O98

[a] Figures in parentheses indicate values for the outer resolution shell. [b] *R*_merge_=∑_*hkl*_∑_*i*_|*I_i_*(*hkl*)−⟨*I*(*hkl*)⟩|/∑_*hkl*_∑_*i*_ *I_i_*(*hkl*). [c] *R*_meas_=∑_*hkl*_ [*N*/(*N*−1)]^1/2^×∑_*i*_|*I_i_*(*hkl*)−⟨*I*(*hkl*)⟩|/∑_*hkl*_∑_*i*_ *I_i_*(*hkl*), where *I_i_*(*hkl*) is the *i*th observation of reflection *hkl*, ⟨*I*(*hkl*)⟩ is the weighted average intensity for all observations *i* of reflection *hkl*, and *N* is the number of observations of reflection *hkl*. [d] *CC* ${}_{1/2}$ is the correlation coefficient between intensities taken from random halves of the dataset. [e] The data set was split into “working” and “free” sets consisting of 95 and 5 % of the data, respectively. The free set was not used for refinement. [f] The *R* factors *R*_work_ and *R*_free_ are calculated as follows: *R*=∑(|*F*_obs_−*F*_calc_|)/∑|*F*_obs_|, where *F*_obs_ and *F*_calc_ are the observed and calculated structure factor amplitudes, respectively. [g] As calculated by using MolProbity.^19^

The two subunits of VAS in the asymmetric unit form a dimeric assembly. Part of the interface involves an extended loop from each subunit (residues 103–124), delineated by β5 and β6 that form a β‐hairpin, although sections of this loop are disordered in both subunits (Figure 3); this is referred to as the “flap domain” in related structures. The core of each subunit is the canonical Rossmann nucleotide binding fold characteristic of the SDR enzymes, in which the cofactor lies across the end of the central, predominantly parallel, β‐sheet. The two active‐site cavities face each other across the apparent dimer interface, with the two opposing NADP^+^ C‐4 atoms only ≈17 Å apart, and are framed by the base of the flap domain and another loop that arises between β8 and α9 (residues 251–266) and folds over the cofactor (i.e., the “cofactor loop”). In this arrangement, the active centres are effectively inaccessible to bulk solvent, thus prompting us to speculate that the quaternary structure suggested by the crystal structure is not representative of the active state of the enzyme. In support of this proposal, the elution profiles of VAS in size exclusion chromatography and dynamic light scattering analysis are consistent with a monomeric species with an estimated molecular size of 32 kDa (calculated molecular mass of His_6_‐VAS is 34 469 Da, Figure S11). The structurally related plant SDRs SalR, MNMR and IPR are also reported to be monomeric, and they all superimpose closely on the VAS structure with overall RMSDs of only 1.2–1.3 Å for main‐chain atoms. Nevertheless, there is significant variation in the length and conformation of the flap domain (Figures 3 and S10), which generally shows elevated temperature factors relative to the rest of the protein, implying some flexibility in this region.

Whereas in the closely related enzymes this flap is folded over the substrate‐binding pocket, in VAS the flap adopts a rather open conformation, but this conformation is most probably determined by the crystal contacts described above. Thus, it seems likely that the flap is a dynamic motif, perhaps playing a role in substrate binding and, furthermore, might be important in shielding the site of reduction from bulk solvent. In the more distantly related human carbonyl reductase (CBR1; PDB ID: 1WMA;^18^ rms deviation vs. VAS of 1.8 Å), the equivalent of the flap domain is a short loop that is unlikely to impinge on the active site at all.

It is clear that, when viewed as a molecular surface, the cofactor loop is implicated in capturing the NADP^+^, as well as in creating one side of the substrate‐binding pocket (Figure 3 C and D). This is consistent with an ordered “bi–bi” mechanism, in which the reduced cofactor binds first and the oxidized cofactor leaves last. The substrate‐binding pocket itself is comparatively constricted, with the C‐4 atom of the nicotinamide ring projecting into it. By allowing a degree of protein side‐chain flexibility (specifically to Ile168, Met169, Phe220, Phe253 and Asn259) in docking simulations, it was possible to obtain a putative ternary complex that placed the hydride acceptor atom (C‐17) of the substrate within 4 Å of the nicotinamide C‐4 atom (with a binding affinity of −7.0 kcal mol^−1^). Although this distance is slightly larger than needed for hydride transfer, this showed that the dimensions of the substrate are comparable to those of the active‐site pocket (Figure 3 D). This exercise provides some insight into the structure of the ternary complex but in view of the flexibility of the active‐site flap, the loop and the substrate itself the full interactions between VAS and its substrate cannot be predicted or simulated. Unfortunately, neither substrate nor product could be co‐crystallized with the enzyme.

### Mutations in the VAS active‐site triad

**Figure 4:**
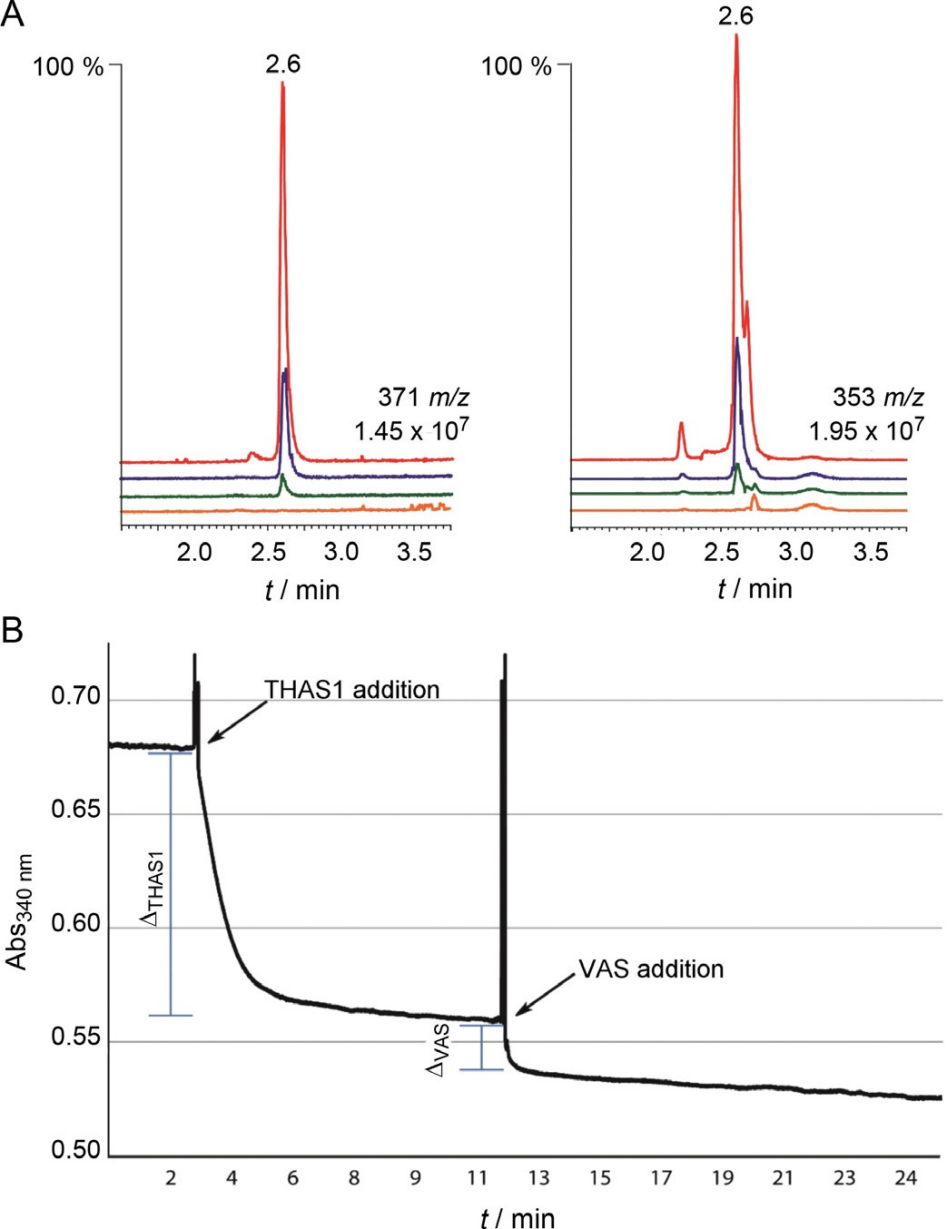
Biochemical assay of VAS. A) LC‐MS chromatograms showing product formation of VAS wild type (Cro013448, red) and its mutants Y223A (orange), S167A (blue) and K227A (green). LC‐MS chromatograms of the hydrated (371 *m*/*z*) and the dehydrated product (353 *m*/*z*) on the left and right, respectively. B) Typical spectrum of NADPH absorbance at 340 nm during reduction of strictosidine aglycone (100 μm) by THAS1 and VAS (Cro013448) at pH 6. The addition of VAS was after THAS1 had reduced its available substrate (plateau). VAS further reduced strictosidine aglycone, thus indicating that these two enzymes do not compete for the same substrate.

To explore the mechanism of action of VAS, the amino acids Lys227, Ser167 and Tyr223, which form the catalytic triad of typical SDR enzymes,^4^, ^17^ were each mutated to alanine (Figure 4 A). Expression levels of each mutant were similar to that of the wild‐type enzyme. When Tyr223 was mutated to alanine, the major product (*m*/*z* 371 and 353) was no longer produced, thus indicating that the active‐site tyrosine residue is necessary for catalysis, which is typical for SDR enzymes.^4^ Mutation to Lys227 and Ser167 did not have a dramatic impact on the product profile, although they both qualitatively reduced the enzyme activity as determined by endpoint assays.

### Kinetic analysis of VAS

Strictosidine aglycone can exist as a variety of isomers, with the equilibrium among these isomers presumably contributing to the diversity of MIA backbones observed in nature (Scheme 2). Little is known about how this equilibrium or numerous keto–enol tautomerism processes are controlled; it is only known that strictosidine aglycone spontaneously rearranges to form a pro‐heteroyohimbine scaffold in the absence of a downstream enzyme (Scheme 2).^12^ Kinetic analysis of VAS by spectrophotometric methods was attempted, but initial assays showed the rapid formation of only very small amounts of product. This suggested that the strictosidine aglycone isomer that serves as the substrate for VAS (pro‐vitrosamine, **11**, Figure 3) only represents a small portion of the total strictosidine aglycone pool (<5 %). This, combined with the low solubility of strictosidine aglycone in water, meant that carrying out meaningful steady‐state kinetic analyses of VAS was not possible.

However, it was possible to calculate the relative abundance of pro‐vitrosamine relative to a major strictosidine aglycone isomer—pro‐heteroyohimbine. Several medium‐chain alcohol dehydrogenases that reduce the pro‐heteroyohimbine isomer to form heteroyohimbines have been discovered recently.^10^, ^20^ To compare substrate abundances, strictosidine aglycone was allowed to equilibrate in solution for 10 min at room temperature in order to generate all its isomers. After this, strictosidine aglycone was first reduced by THAS1, a heteroyohimbine synthase, which allowed the estimation of the abundance of pro‐heteroyohimbine in solution. THAS1 consumed the available substrate (pro‐heteroyohimbine) within 5 min. Subsequent addition of VAS resulted in further consumption of strictosidine aglycone (Figure 4 B), thus suggesting that THAS1 and VAS do not compete for the same substrate. The difference in absorbance from THAS1 addition to the plateau (Δ_THAS1_) was calculated in triplicate and was determined to be (16.5±2.5) % of the total strictosidine aglycone substrate pool. The same was done for VAS, and it was determined that the pro‐vitrosamine represents (3.1±0.7) % of the total strictosidine aglycone pool. Similar results were obtained when VAS was incubated with strictosidine aglycone prior to THAS1 addition. These data suggest that the equilibrium among the different strictosidine aglycone isomers occurs on a very slow timescale.

## Conclusion

The extensive chemical diversity of the MIAs is largely derived from the reactivity of the strictosidine aglycone substrate. Two previously reported dehydrogenases that use strictosidine aglycone as a substrate turn over specific strictosidine aglycone isomers—pro‐heteroyohimbine or dehydrogeissoschizine—to generate highly divergent structures^20^, ^21^ (Scheme 2). Although the experiments reported here do not unequivocally demonstrate that strictosidine aglycone is the physiological substrate, the in vitro reactivity of VAS clearly shows that a third isomer of strictosidine aglycone can be reductively trapped. A reductase that can trap this alternative strictosidine aglycone isomer has never been previously identified.^22^, ^23^, ^24^

The reduction catalysed by VAS probably works by a standard SDR mechanism, with the C‐4 hydride of NADPH attacking C‐17 of the iminium substrate. As previously reported for other SDRs, the resulting intermediate, which might be polarized by Ser167, would then be protonated by Tyr223, which might have a reduced p*K*_a_ value due to interactions with the basic side chain of Lys227 and the hydrogen bonding network with the cofactor ribose moiety. VAS could bind the correct strictosidine aglycone isomer in solution or, alternatively, catalyse its formation from another strictosidine aglycone isomer in the enzyme active site. In silico docking suggests that the substrate is oriented in a way that promotes hydride transfer to the correct face of pro‐vitrosamine. The weak electron density of the flap that covers the active site indicates that it is mobile, so it is difficult to understand exactly how VAS selectively binds and reduces the pro‐vitrosamine isomer. Kinetic experiments (Figure 4 B) suggest that the enzyme binds the correct isomer from solution, in which it is present in very low levels. Pro‐heteroyohimbine, which is the substrate for heteroyohimbine synthases such as THAS1, makes up approximately 16 % of the strictosidine aglycone pool whereas the VAS substrate represents just 3 %. The inherent reactivity and equilibration of the different forms of strictosidine aglycone might play a role in establishing the most abundant alkaloid species in plants that can be utilized and built upon.

The discovery of VAS demonstrates the capacity of an SDR to reduce an additional isomer of strictosidine aglycone to form a new alkaloid backbone. The product of VAS, vitrosamine, has never been described in the literature. Vitrosamine may be a “cryptic” natural product: one that is only produced by the plant under certain conditions. Alternatively, vitrosamine might be an “unnatural natural product”: a product that is not formed in vivo but is observed in vitro, from which it draws its name. This discovery highlights the potential of plant genome mining for the discovery of new biosynthetic enzymes, as is being done extensively with biosynthetic gene clusters from microorganisms. This enzyme adds to the biosynthetic toolkit available for heterologous expression and the production of both natural and “unnatural natural” products. This discovery also expands the already large chemical diversity possible from the central MIA precursor, strictosidine.

## Experimental Section

**Cloning of Cro013448 and Rse2785**: Sequence data for *C. roseus* and *R. serpentina* were obtained from the Medicinal Plant Genomics database (http://medicinalplantgenomics.msu.edu/). Cro013448 was cloned from *C. roseus* cDNA by using primers with overhangs (5′‐AAGTT CTGTT TCAGG GCCCG GCCGC CATGG GTACC and 3′‐ATGGT CTAGA AAGCT TTATT CAAAC GATGA CTCCT CGC) for directional cloning into the *E. coli* expression vector pOPINF.^25^ *R. serpentina* Cro013488 homologue (Rse2785) was also cloned into pOPINF after amplification from *R. serpentina* cDNA by using primers with overhangs (5′‐AAGTT CTGTT TCAGG GCCCG TTGAG TAATA CATCC GTCAT and 3′‐ATGGT CTAGA AAGCT TTACT CAAAC GATGA CTCCT CAC). Amplified gene fragments were ligated into pOPINF vector by using the In‐Fusion kit (Clontech–Takara, Mountain View, CA, USA). Positive colonies were selected on LB agar plates supplemented with carbenicillin (100 μg mL^−1^) and checked by PCR with the gene‐specific primers. Identities of the inserted sequences were confirmed by Sanger sequencing.

**Expression of Cro013448 and Rse2785**: Proteins were expressed in SoluBL21 *E. coli* cells (Novagen, Merck Millipore). Cells were grown overnight, and cultures were diluted 1:100 in 2×YT medium (100 mL) supplemented with carbenicillin (100 μg mL^−1^). Protein expression was induced when the cultures reached OD_600_=1 by using isopropyl β‐d‐thiogalactopyranoside (IPTG, 0.1 mm). After 3.5 h incubation at 37 °C the cultures were chilled on ice and centrifuged to harvest the cells. The pellet was washed with PBS and stored at −80 °C overnight. After thawing and re‐suspending in buffer A [100 mL, Tris**⋅**HCl (pH 8, 50 mm), glycine (50 mm), NaCl (500 mm), glycerol (5 %), imidazole (20 mm), 2‐mercaptoethanol (2 mm)] with EDTA‐free protease inhibitor (Roche Diagnostics), cells were lysed by sonication and the cell debris were pelleted by centrifugation (17 000 *g*, 20 min). His_6_‐tagged enzymes were purified with an ÄKTAxpress purifier (GE Healthcare) and on a HisTrap FF 5 mL column (GE Healthcare) equilibrated with Buffer A. Samples were loaded at a flow rate of 4 mL min^−1^ and step‐eluted with buffer B [Tris**⋅**HCl (pH 8, 50 mm), glycine (50 mm), NaCl (500 mm), glycerol (5 %), imidazole (500 mm), 2‐mercaptoethanol (2 mm)]. Eluted proteins were subjected to further purification on a Superdex Hiload 26/60 S75 gel filtration column (GE Healthcare) at a flow rate of 3.2 mL min^−1^ with buffer C [HEPES (pH 7.5, 20 mm), NaCl (150 mm), 2‐mercaptoethanol (2 mm)] and collected in 8 mL fractions. The fractions were analysed by SDS‐PAGE, and those containing no traces of other contaminating proteins were pooled. Proteins were concentrated in a 10 kDa cutoff Millipore filter (Merck Millipore) and buffer‐exchanged into buffer C supplemented with TCEP (0.5 mm). The protein concentrations were measured spectroscopically by using the calculated MW and extinction coefficients (21 680 and 24 660 L mol^−1^ cm^−1^, respectively). The His‐tag was not cleaved prior to use of the protein sample. Dynamic light scattering (DLS) was used to monitor the solution properties of the purified sample with a DynaPro‐Titan molecular‐sizing instrument at 25 °C (Wyatt Technology, Haverhill, UK) with use of 13 μL of sample at a concentration of approximately 10 mg mL^−1^.

**Enzyme assays**: Strictosidine (300 μm) was incubated with purified SGD (10 nm) in citrate buffer (pH 6.0, 50 mm) for 15 min at room temperature. NADPH (500 μm) was added, followed by purified Cro013448 or Rse2785 (1 μm), and the reaction mixtures were mixed and incubated for 10 min at room temperature. The reaction was stopped by addition of 1 volume of methanol, and the mixture was vortexed and centrifuged at 17 000 *g* for 10 min. An aliquot (20 μm) was mixed with mobile phase (80 μm) and analysed with a UPLC‐MS (Waters) equipped with an Acquity BEH C18 1.7 μm 2.1×50 mm column connected to a Xevo TQS mass spectrometer (Waters). Product detection was done as previously described,^19^ and a separation method described previously was used (Method 2 for VAS and Method 1 for mutants).^19^

**Spectroscopic enzyme assays**: Strictosidine (100 μm) was deglycosylated by using SGD (10 nm) at 30 °C in a spectrophotometer cuvette in citrate buffer (pH 6.0, total volume of 800 μL, 50 mm). The completion of the reaction was verified by mass spectrometry. NADPH (100 μm) was added to the reaction mixture and mixed by pipetting. The reaction was monitored at 340 nm with a spectrophotometer (Cary 50 Bio, Varian) at room temperature. After verification that the NADPH absorbance was stable, purified THAS1 (1 μm) was added and mixed. The reaction was allowed to progress until the plateau was reached, and then Cro013448 (1 μm) was added to the reaction and mixed and the reaction was allowed to reach equilibrium. The difference in NADPH (ΔABS_340_) was recorded on a UV/Vis spectrophotometer (Cary WinUV Kinetics, Application v.3.00(182), Varian) before addition of each enzyme, and at the point at which the reaction reached the plateau. The assay was done in triplicate. The inverse reaction (Cro013448 added first, then THAS1) was also carried out.

**Vitrosamine synthesis and isolation**: Strictosidine (30 mg) was incubated in a final volume of 100 μL (final concentration 566 μm) with citrate buffer (pH 6, 50 mm) and purified SGD (10 nm) to generate strictosidine aglycone. NADPH (700 μm) and Cro013448 (3 μm) were added and the reaction mixture was incubated at 37 °C with gentle shaking. After 2 h, more Cro013448 was added (concentration brought to 6 μm). The reaction was stopped after 4 h by addition of NaOH to approximately pH 9.5 and the product was extracted with EtOAc (5×20 mL). The EtOAc fraction was dried under vacuum, re‐suspended in EtOAc (100 μL) and loaded onto a nano‐silica TLC [pre‐basified with triethylamine (TEA)]. The TLC was run in EtOAc/hexanes/TEA (50:50:1), and the product band was excised from the silica, extracted with EtOAc (50 mL), filtered and dried under vacuum.

**Synthesis of deuterium‐labelled vitrosamine**: Deuterated Cro013448 product was produced by using deuterated cofactor (NADPD). Strictosidine (15 mg) was incubated in HEPES buffer (pH 7.0, 50 mm) with NADP^+^ (200 μm) in a total volume of 141 mL. NADPD was generated in situ by using [1‐^2^H]‐d‐glucose (250 μm, Cambridge Isotope Laboratories, Inc., USA) and glucose dehydrogenase (800 U, from *Pseudomonas* sp., Sigma–Aldrich) as an NADPH regeneration system. Purified SGD and Cro013448 (1 μm final concentration) were then added and the mixture was incubated at 31 °C for 16 h with gentle shaking. The reaction was stopped by addition of saturated NaOH. The product was extracted multiple times in a total volume of 120 mL of EtOAc and dried. The labelled product was purified from TLC as described above.

**Cro013448 enzyme product characterization**: For high‐resolution MS analysis, purified Cro013448 product was infused at 5–10 μL min^−1^ into a Synapt G2 HDMS mass spectrometer (Waters) calibrated by use of a sodium formate solution. The sample was analysed for 2 min with a scan time of 1 s in the range of 50–600 *m*/*z*. Capillary voltage was 3.5 V, cone voltage 40 V, source temperature 120 °C, desolvation temperature 350 °C, desolvation gas flow 800 L h^−1^. Leu‐enkephalin peptide (1 ng μL^−1^) was used to generate a dual lock‐mass calibration with [*M*+H]^+^=556.2766 and *m*/*z* 278.1135 measured every 10 s. For MS^2^, the precursor ion of *m*/*z* 371 was selected and fragmented with a collision energy of 20 V. The mass of the product was found to be 353.1862 *m*/*z* and 371.1967 *m*/*z* for the two major species present in solution.

For NMR analysis, the product was dissolved in CDCl_3_. 1D ^1^H NMR, ^1^H,^1^H COSY, HMQC and HMBC were recorded with a Bruker Avance III HD 700 NMR spectrometer (16.4 T, ^1^H operating frequency 700 MHz) equipped with TCI H‐C/N‐D 1.7 mm microcryoprobe. The deuterium‐labelled product was re‐suspended in CDCl_3_ (500 μL), and the 1D ^1^H and 2D ^1^H,^1^H COSY spectra were measured with a Bruker Avance NMR spectrometer operating at 400 MHz for ^1^H and equipped with a BBFO plus 5 mm probe.

**Crystallization and data collection**: Purified His_6_‐VAS (≈10.8 mg mL^−1^) was used for crystallization trials in MRC2 96‐well sitting‐drop vapour diffusion crystallization plates (Swissci) with a mixture of well solution (0.3 μL) and protein solution (0.3 μL). After initial hits with commercial screens, optimization screens were set up by use of an Oryx8 robot (Douglas Instruments, Hungerford, UK). The best crystals were obtained from MMT (malic acid/MES/Tris) buffer (pH 4.0, 0.1 m) and PEG 3350 (24 %, *w*/*v*), from plates incubated at 20 °C. Crystals were cryoprotected with well solution containing ethylene glycol (25 %) for ≈1 min and flash‐cooled in liquid nitrogen.

Crystals were transferred robotically to the goniostat on beamline I02 at the Diamond Light Source (Oxfordshire, UK) and maintained at −173 °C with a Cryojet cryocooler (Oxford Instruments). X‐ray diffraction data were recorded with a Pilatus 6M detector (Dectris, Baden‐Dättwil, Switzerland) and were then integrated by using XDS^26^ and scaled and merged by using AIMLESS^27^ with the XIA2 expert system.^28^ The best data set was processed to a resolution of 1.55 Å in space group *P*2_1_2_1_2_1_, with approximate cell parameters of *a*=59, *b*=61, *c*=162 Å (see Table 1 for a summary of data collection statistics).

The structure was solved by molecular replacement by using PHASER^29^ and the crystal structure of SalR (PDB ID: 3O26^15^), with which it shares 53 % amino acid sequence identity, as template. The asymmetric unit contained two monomers, corresponding to an estimated solvent content of 44 %. After refinement of this preliminary model by using REFMAC5,^30^ phases were improved through density modification (incorporating twofold averaging) in PARROT,^31^ to yield a much improved electron density map at 1.55 Å resolution. This enabled a new model of VAS to be built from scratch by using BUCCANEER,^32^ and this was completed by several iterations of manual rebuilding in COOT^33^ and restrained refinement in REFMAC5 with use of isotropic thermal parameters and TLS group definitions obtained from the TLSMD server (http://skuld.bmsc.washington.edu/∼tlsmd).^34^ Model geometries were validated with MolProbity^19^ before submission to the Protein Data Bank (see Table 1 for a summary of model statistics). An omit *mF*_obs_−*dF*_calc_ difference electron density map was generated for the bound NADP^+^ with use of phases from the final model without the cofactor after the application of small random shifts to the atomic coordinates, resetting of temperature factors, and re‐refining to convergence (Figure 3 E). All structural figures were prepared by using CCP4MG.^35^

**Docking simulations**: A model of a ternary complex consisting of VAS, cofactor and substrate was obtained by docking simulations by using AutoDock Vina.^36^ The VAS X‐ray structure comprised the “receptor”, its oxidized cofactor was substituted by NADPH, and all solvent molecules were removed. Coordinates (including hydrogen atoms) for the “ligand” (i.e., the substrate) were generated by using the Lidia tool^37^ within COOT. Receptor and ligand were prepared for the docking simulations by using AutoDockTools^38^ in PDBQT format. Initial runs with a fully rigid receptor did not allow the close approach of the ligand to the NADPH. Therefore, in subsequent runs, flexibility was allowed in the side chains of selected residues in the vicinity of the expected substrate‐binding site. AutoDock Vina was run with a box size of 18×18×18 Å covering the active‐site pocket and an “exhaustiveness” value of 64; otherwise the default settings were used.

**Active‐site mutations**: The three catalytic residues were each mutated to alanine through codon mutations (S167A, Y223A, K227A). Mutant Cro013448 gene fragments were obtained from Integrated DNA Technologies, Coralville, Iowa, USA with the pOPINF overhangs included. Ligation into pOPINF and clone selection were performed with the gene‐specific primers as described above; sequences were verified by Sanger sequencing. Small‐scale expression of mutants (50 mL) was performed at 37 °C as described above.

## Conflict of interest

*The authors declare no conflict of interest*.

## Supporting information

As a service to our authors and readers, this journal provides supporting information supplied by the authors. Such materials are peer reviewed and may be re‐organized for online delivery, but are not copy‐edited or typeset. Technical support issues arising from supporting information (other than missing files) should be addressed to the authors.

Supplementary
