# Supplementary material for: Discovery of a Short‐Chain Dehydrogenase from Catharanthus roseus that Produces a New Monoterpene Indole Alkaloid
Source: Chembiochem. 2018 Mar 22;19(9):940–8. doi: 10.1002/cbic.201700621 (PMC6003104; doi:10.1002/cbic.201700621)
Supplement: Supplementary file 1 — Supplementary [file CBIC-19-940-s001.pdf]

CHEMBIOCHEM

Supporting Information

Discovery of a Short-Chain Dehydrogenase from *Catharanthus roseus* that Produces a New Monoterpene Indole Alkaloid

Anna K. Stavrinides,[a, b] Evangelos C. Tatsis,[a] Thu-Thuy Dang,[a] Lorenzo Caputi,[a]
Clare E. M. Stevenson,[a] David M. Lawson,[a] Bernd Schneider,[c] and Sarah E. O'Connor*[a]

cbic_201700621_sm_miscellaneous_information.pdf

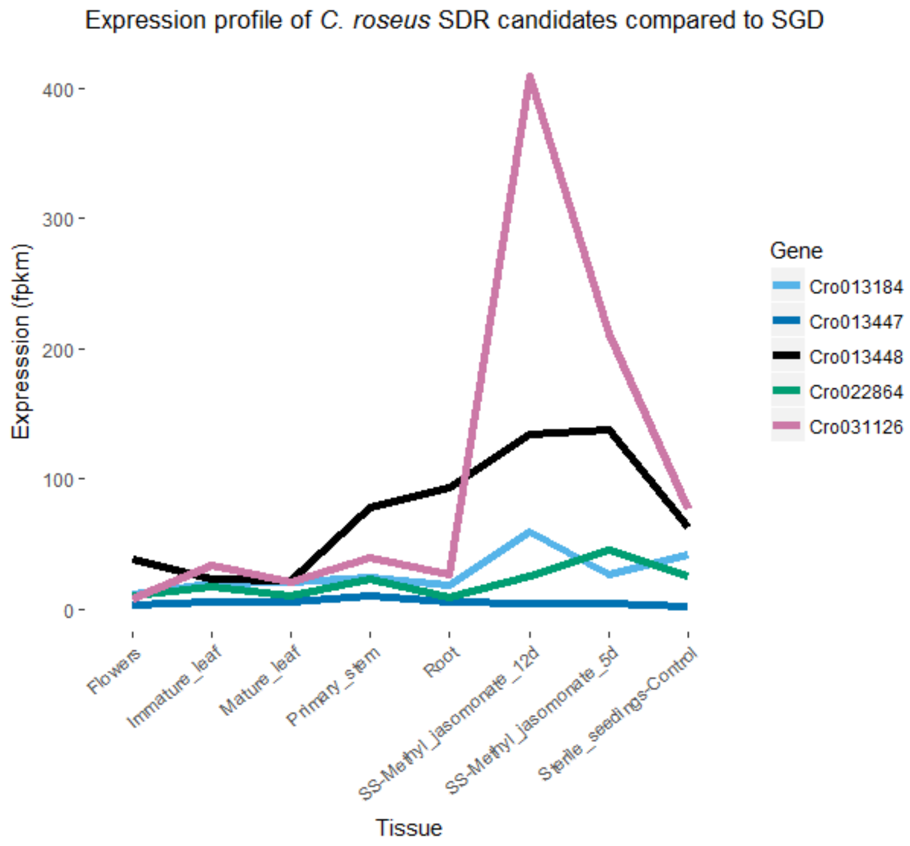

Supporting Figure 1. Expression profile of VAS (Cro013448, in black)) in different tissues compared to the expression profile of other candidate SDRs (Cro013184, Cro013447, Cro022864) which did not reduce strictosidine aglycone in vitro, and Strictosidine Glucosidise (SGD, Cro031126, in pink). VAS and SGD have a high expression in seedlings (Sterile_seedlings-Control) and are upregulated at 5 and 12 days after treatment with Methyl Jasmonate (SS-Methyl_jasmonate_5d, and SS-Methyl-jasmonate_12d). Cro013184 and Cro022864 were selected as candidates because they showed some upregulation after Methyl Jasmonate treatment; Cro013447 was selected as it is the closest homolog to VAS in *C. roseus*. Expression values collected from Kellner et al. 2015[1].

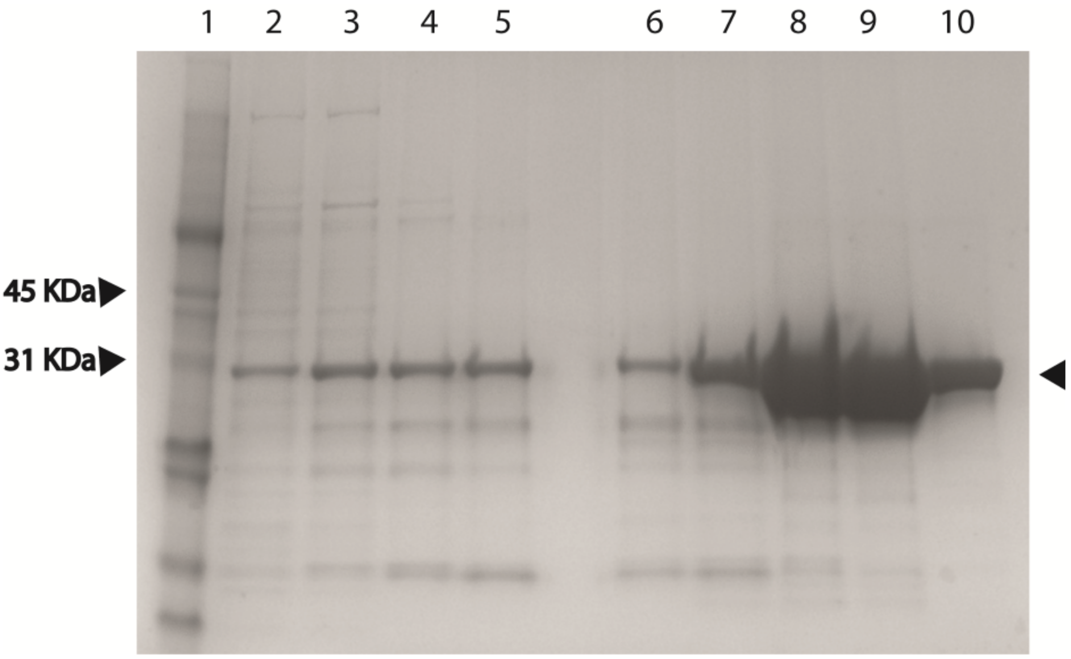

Supporting Figure 2. SDS-PAGE gel of Cro013448. Lane 1: Protein molecular marker; lanes 2-10: elution fractions after two step purification by His-trap and gel-filtration of Cro013448, marked by arrow, expressed in *E. coli*.

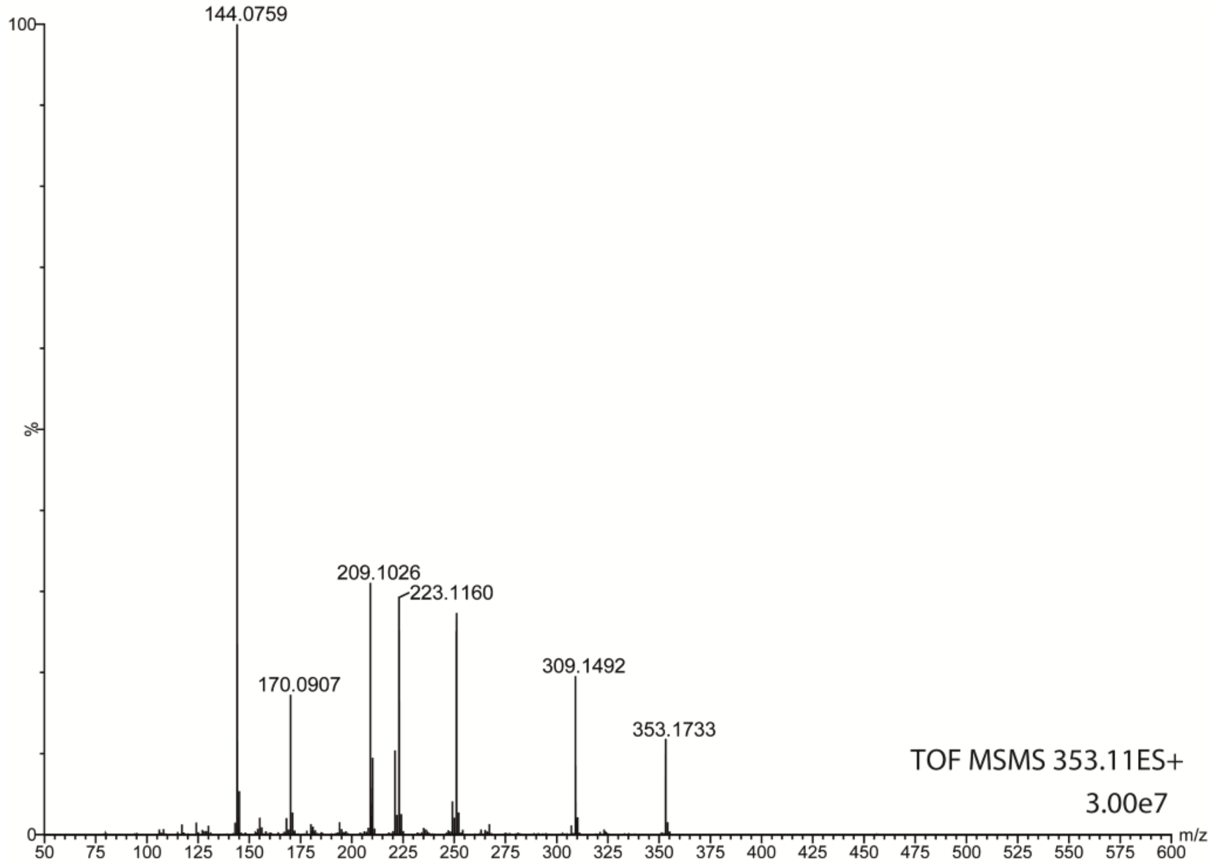

100
%
0
144.0759
209.1026
223.1160
170.0907
309.1492
353.1733
TOF MSMS 353.11ES+
3.00e7
m/z
50 75 100 125 150 175 200 225 250 275 300 325 350 375 400 425 450 475 500 525 550 575 600

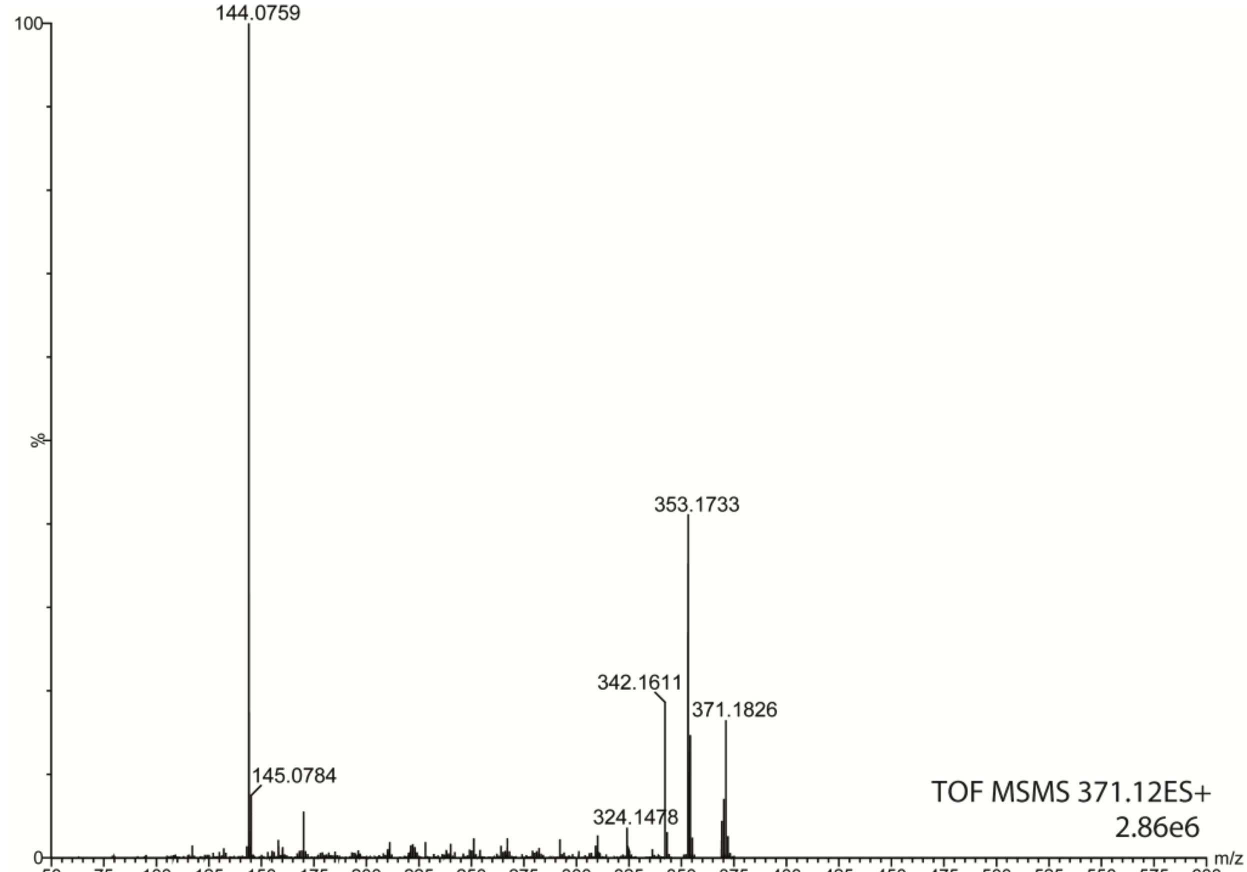

100
%
0
144.0759
353.1733
342.1611
371.1826
145.0784
324.1478
TOF MSMS 371.12ES+
2.86e6
m/z
50 75 100 125 150 175 200 225 250 275 300 325 350 375 400 425 450 475 500 525 550 575 600

Supporting Figure 3. High-resolution MS-MS of the Cro013448 product. The fragmentation of the product produces the fragments 353, 342, 324, and 144.

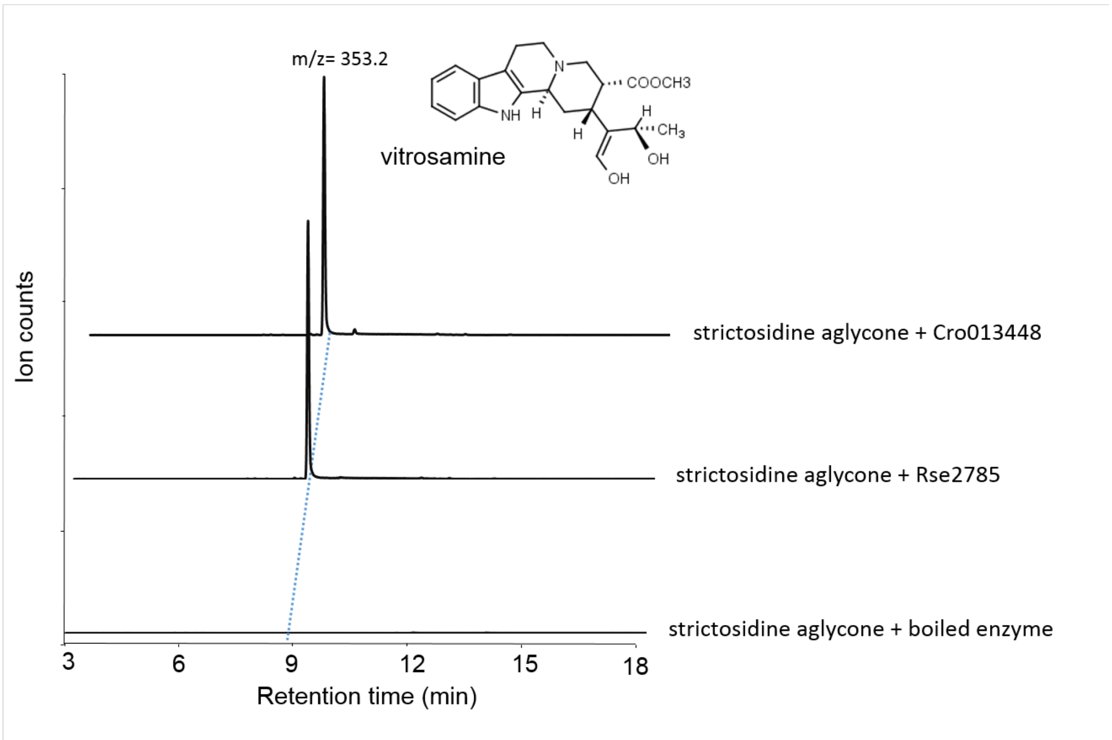

Supporting Figure 4. Cro013448 and its homolog from *R. serpentina* (Rse2785) produce vitrosamine, as determined by co-elution on LC-MS.

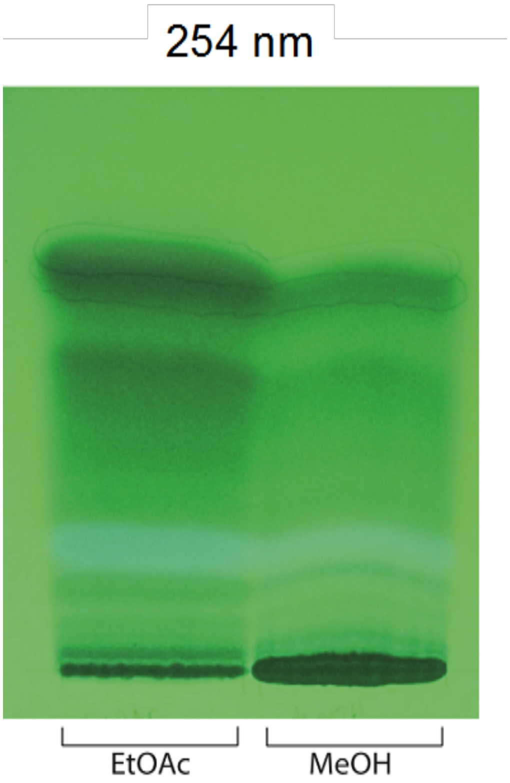

Supporting Figure 5. Large-scale purification of Cro013448 product by preparative thin layer chromatography illuminated with 254 nm. The EtOAc-soluble compounds were spotted on the left and the MeOH-soluble compounds were spotted on the right. The uppermost bands, outlined in pencil, correspond to vitrosamine.

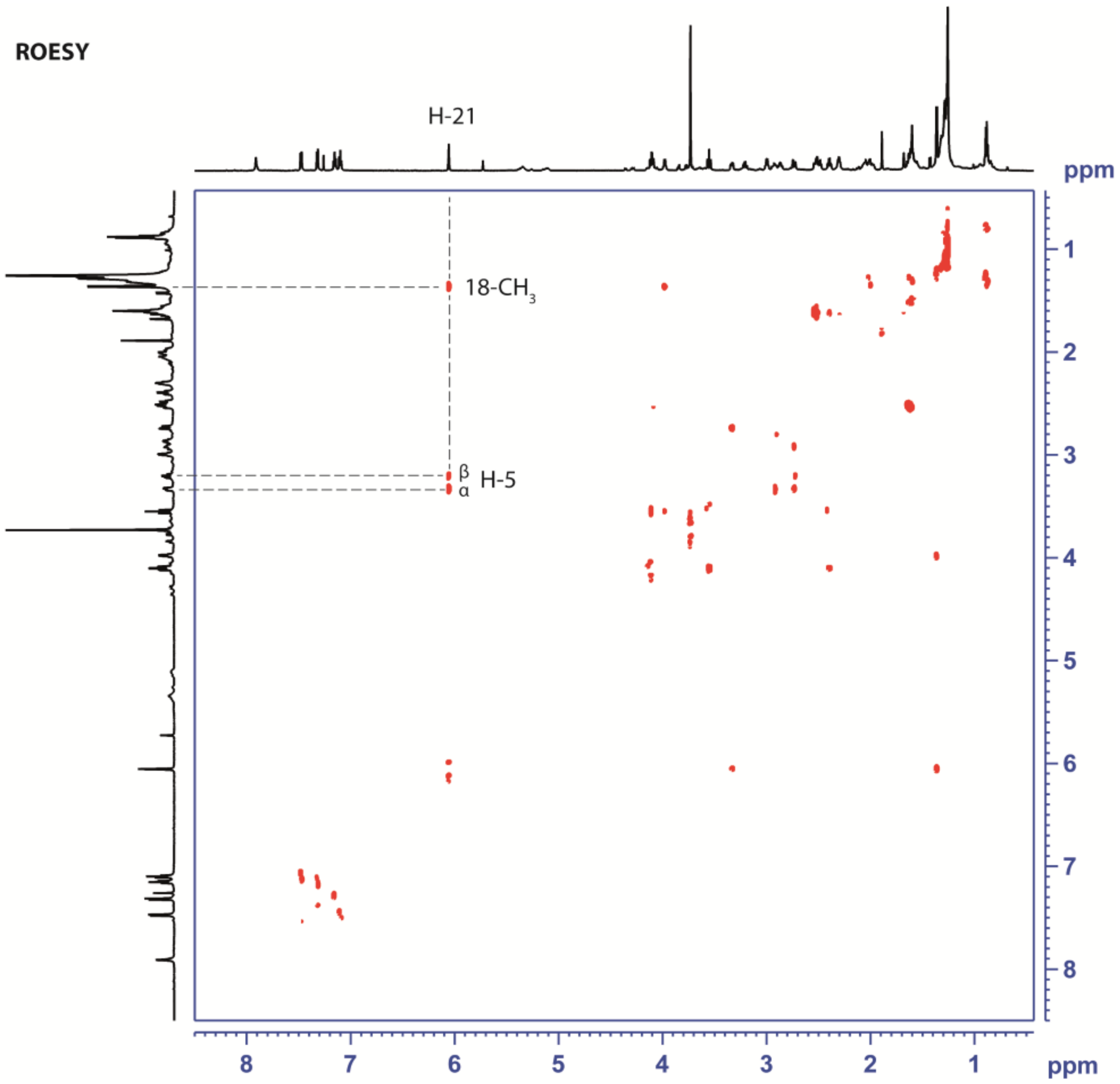

Supporting Figure 6. ROESY spectrum of Cro013448 product. The H-21 is picked out and the correlations with 18-CH_3 and the two H-5 are illustrated in dotted line.

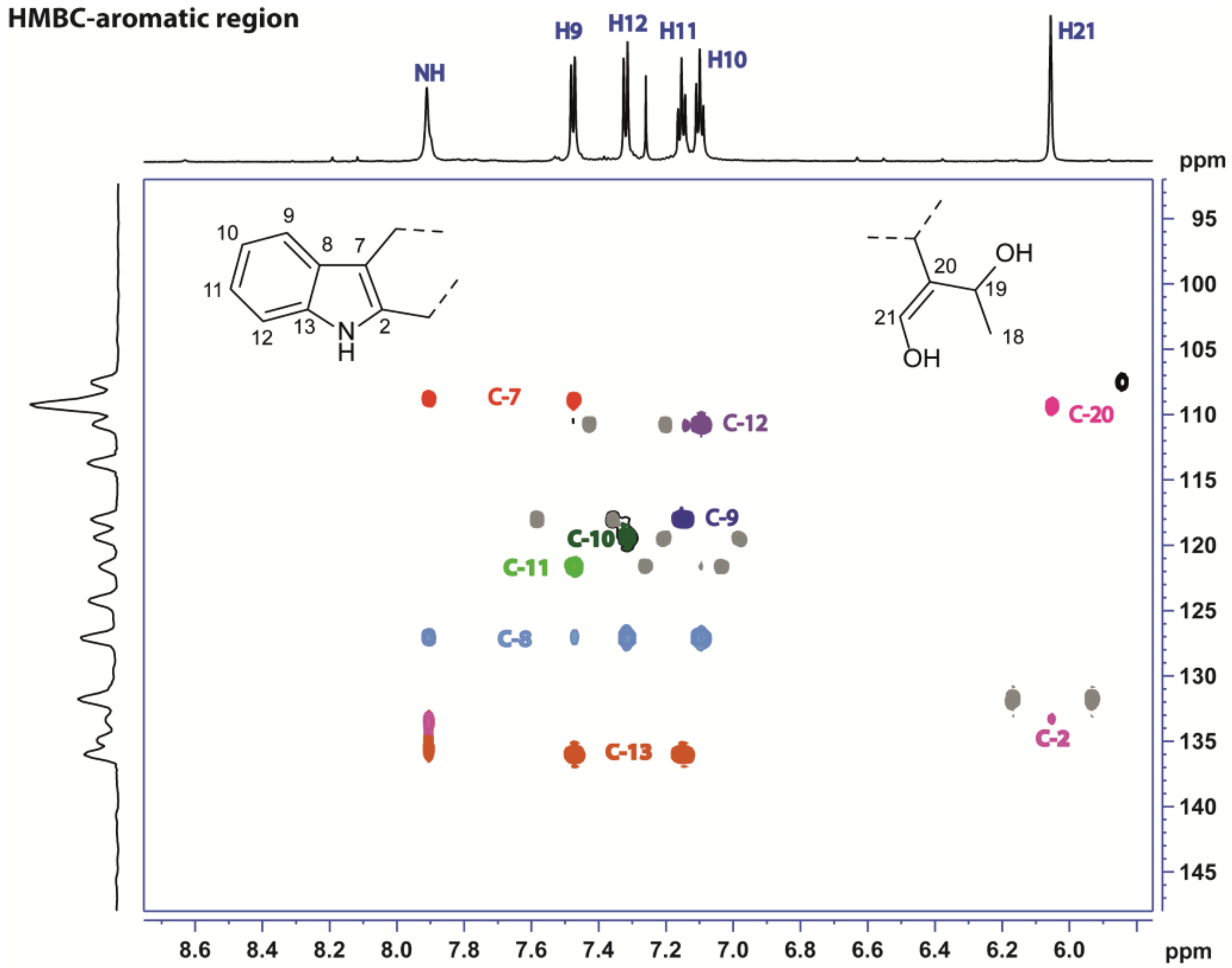

Supporting Figure 7. HMBC spectrum (700 MHz, $CDCl_3$) of Cro013448 product. Display is only of the aromatic region, including H-21. The satellite peaks of carbons are illustrated in grey for clarity. The carbons with correlations to the hydrogens are each coloured differently.

2D NMR

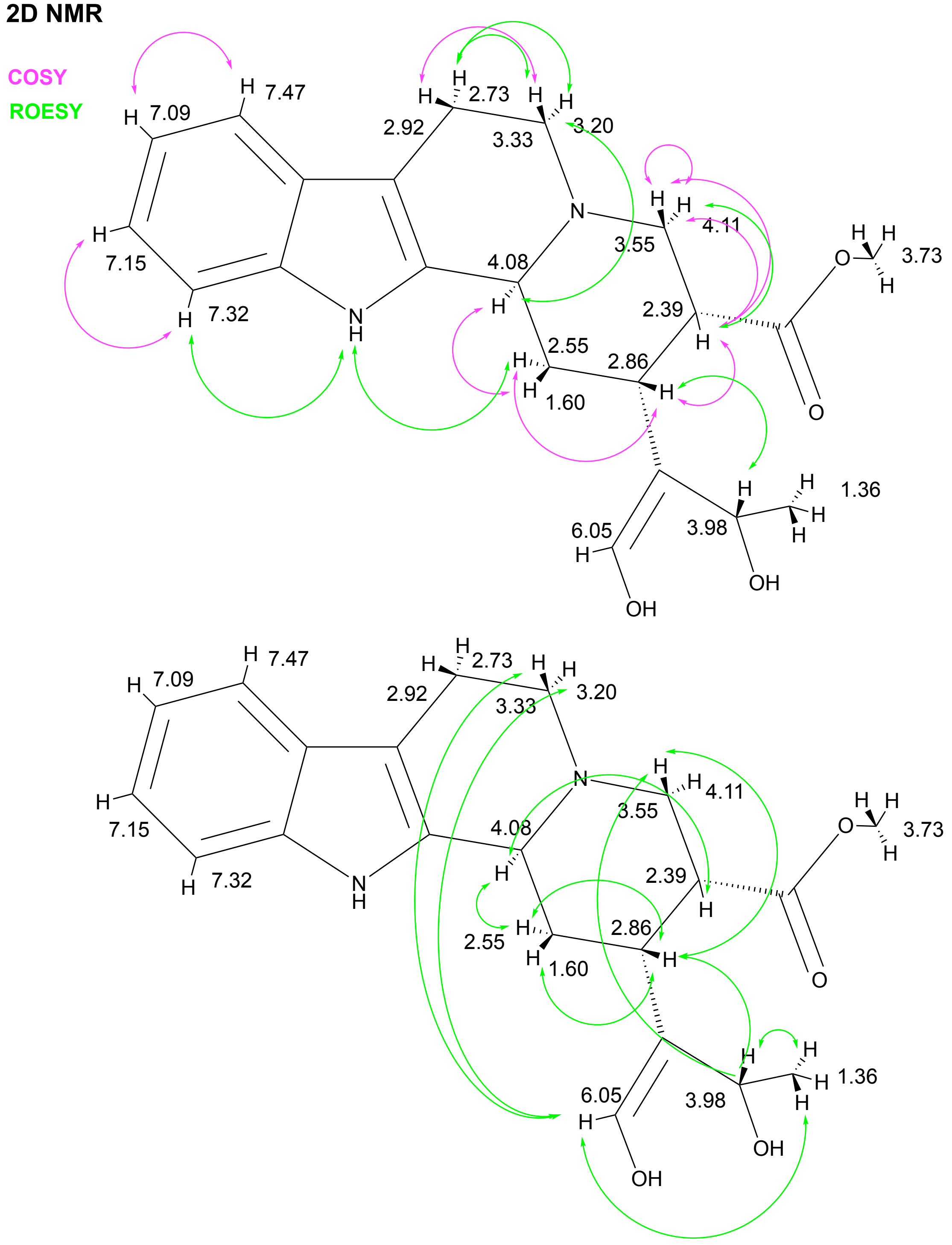

Supporting Figure 8. Correlations between carbons and hydrogens of the Cro013448 product, as measured by NMR (700 MHz) in $CDCl_3$.

2D NMR

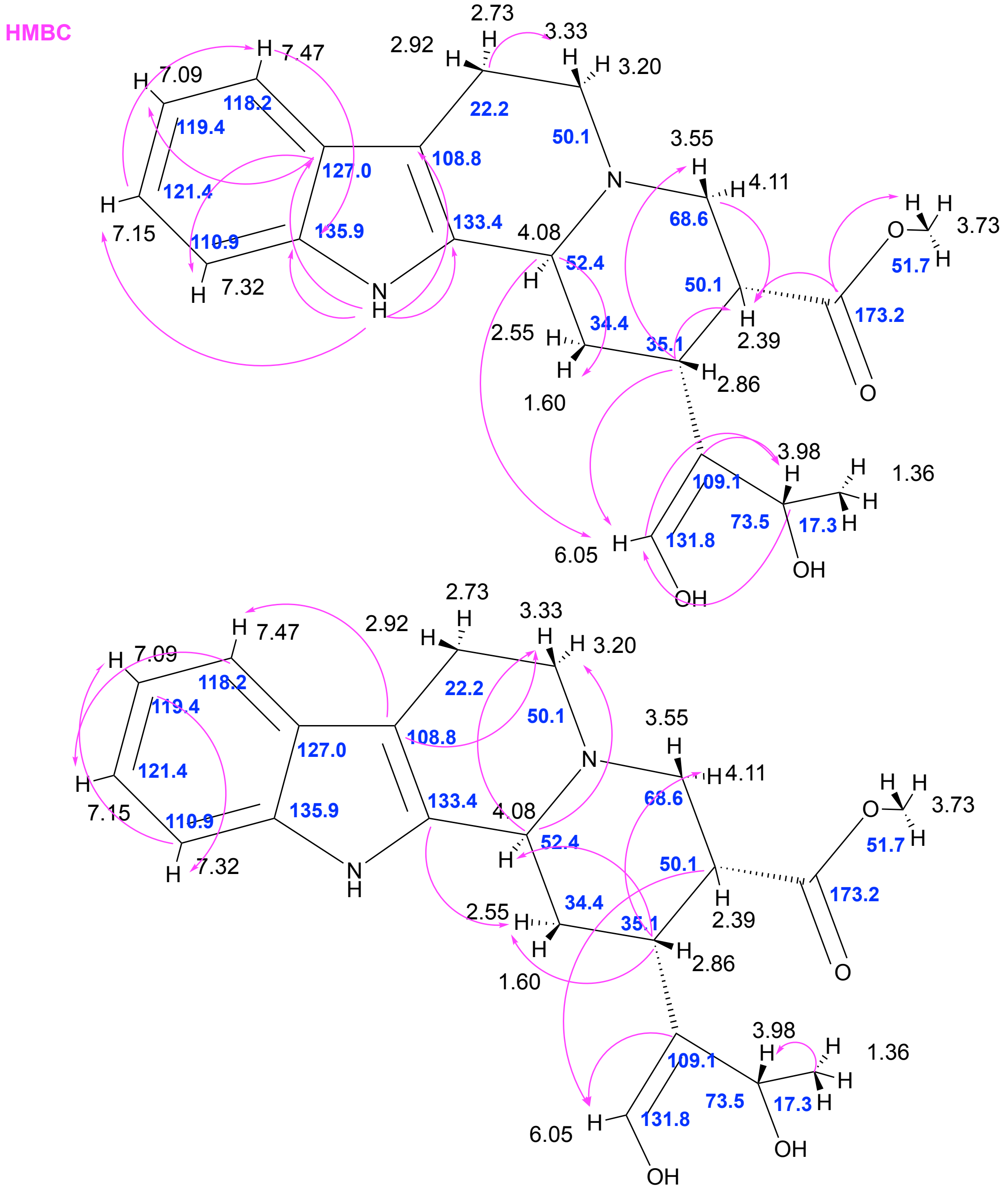

Supporting Figure 9. HMBC correlations between carbons and hydrogens of the Cro013448 product, as measured by NMR (700 MHz) in $CDCl_3$.

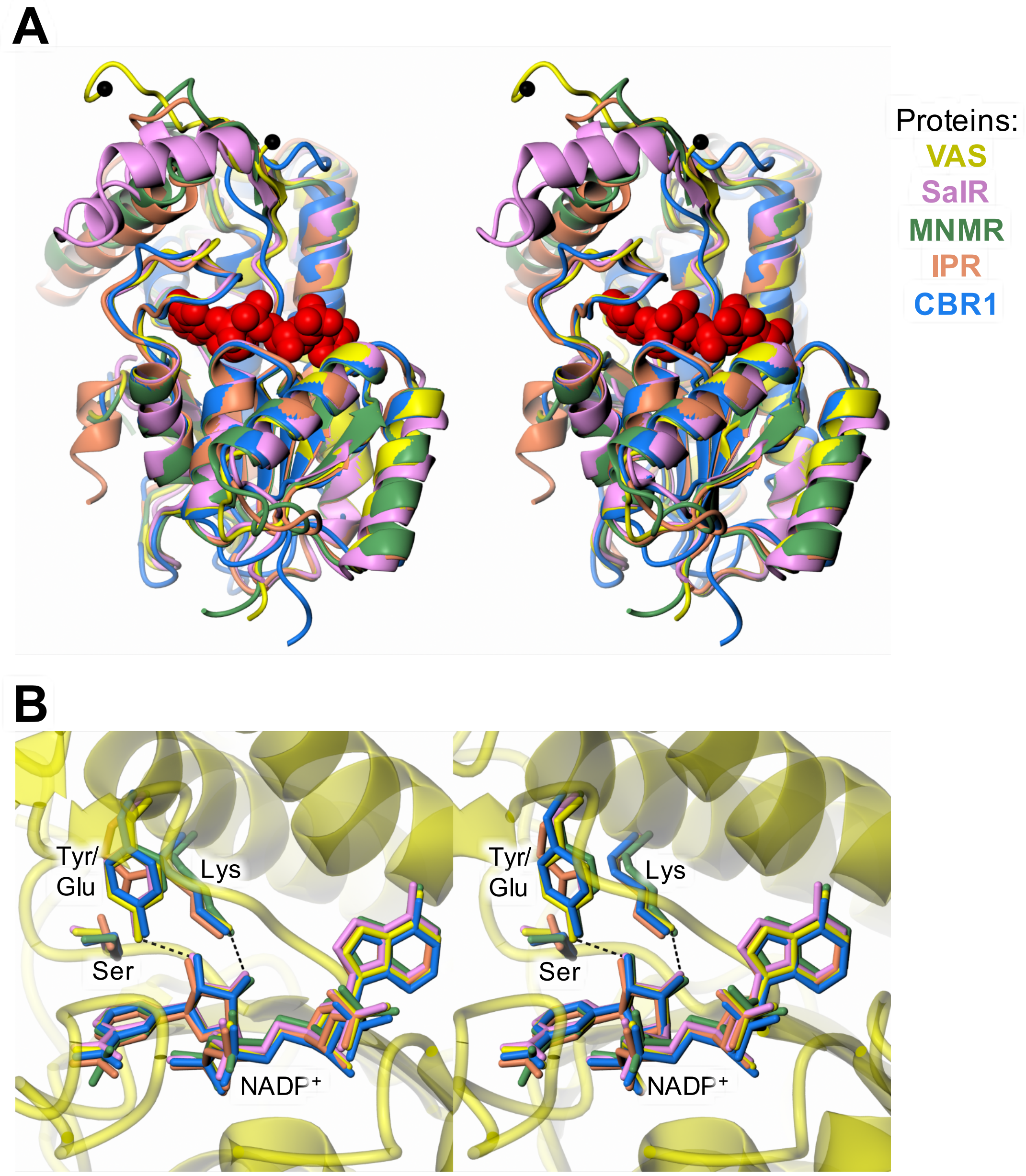

Supporting Figure 10. Superposed X-ray structures of *C. roseus* vitrosamine synthase (VAS, gold; PDB code 5O98), *P. somniferum* salutaridine reductase (SalR, pink; PDB code 3O26), *M. piperita* menthone-neomenthol reductase (MNMR, green; PDB code 5L53), *M. piperita* isopiperitenone reductase (IPR, orange; PDB code 5LCX) and human carbonyl reductase 1 (CBR1, blue; PDB code 1WMA). **A.** Stereoview of the SDRs with black spheres indicating where there is a break in the backbone trace of the flap domain of VAS corresponding to a region that could not be resolved in the electron density. The VAS-bound NADP$^+$ is shown as red van der Waals spheres. **B**. Stereoview of the active sites of the aligned X-ray structures. The

cofactor ($NADP^+$) and the catalytic triad side chains are displayed as cylinders. Interactions between the cofactor ribose and Tyr and Lys are illustrated as dashed lines.

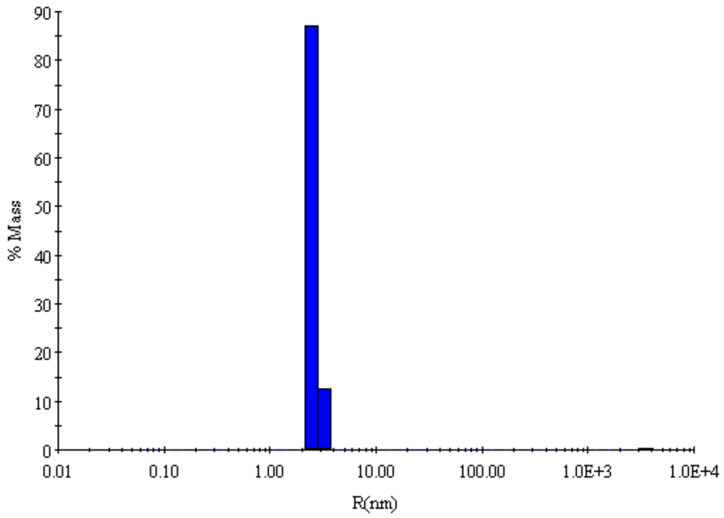

Acq time: 10 s
Num acq: 10
Laser power: 100%
Set temp: 25 °C

Item	R (nm)	%Pd	MW-R (kDa)	%Int	%Mass
Peak 1	2.6	10.2	32	5.4	99.6
Peak 2	43.4	11.3	22790	9.6	0.0
Peak 3	178.9	12.5	627514	56.9	0.0
Peak 4	3619.4	0.0	713950000	28.1	0.3

Supporting Figure 11. Dynamic Light Scattering of Cro013448 demonstrates this protein is a monomer.

Supporting Table 1. ^{1}H (700 MHz) and ^{13}C NMR (135 MHz) data of vitrosamine in $CDCl_3$.

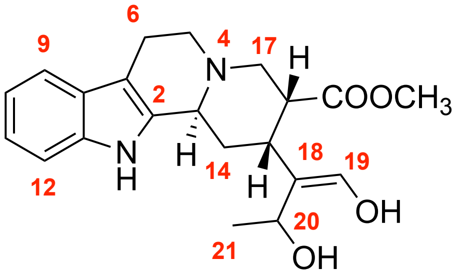

No	^{1}H δ, *mult.*, *J* (Hz)	^{13}C δ
N	7.91 *br*	
2	-	133.6
3	4.08 *m*	52.4
5	3.33 *ddd*, 11.8, 5.6, 2.3<br>3.20 *ddd*, 11.8, 10.8, 4.1	50.1
6	2.92 *ddd* 14.9, 10.8, 5.7<br>2.73 *ddd* 14.9, 4.1, 2.3	22.1
7	-	108.8
8	-	127.0
9	7.48 *d* 7.8	118.2
10	7.10 *dd* 7.8, 7.8	119.4
11	7.15 *dd* 7.8, 7.8	121.7
12	7.32 *d* 7.8	110.7
13	-	136.0
14	1.62 dd 12.8, 10.8<br>2.53 dd 12.8, 6.4	34.3
15	2.87 ddd 10.8, 10.8, 6.4, 1.7	35.1
16	2.40 ddd 11.1, 10.8, 4.4	49.9
17	4.11 dd 11.3, 4.4<br>3.55 dd 11.3, 10.8	68.6
18		109.4
19	6.06 dd 1.7, 1.7	131.8
20	3.98 qd 6.3, 1.7	73.6
21	1.36 d 6.3	17.2
COO		173.2
COOCH$_3$	3.73 s	51.7

Reference:

1. Kellner, F., Kim, J., Clavijo, B.J., Hamilton, J.P., Childs, K.L., Vaillancourt, B., Cepela, J., Habermann, M., Steuernagel, B., Clissold, L. and McLay, K., 2015. Genome-guided investigation of plant natural product biosynthesis. *The Plant Journal*, *82*(4), pp.680-692.
